# Investigating strategies to improve AccesS to Kidney transplantation (the ASK trial): a protocol for a feasibility randomised controlled trial with parallel process evaluation

**DOI:** 10.1186/s40814-023-01241-1

**Published:** 2023-01-20

**Authors:** Pippa K. Bailey, Fergus J. Caskey, Stephanie MacNeill, Rachel Ashford, Lindsay Pryce, Liise Kayler, Yoav Ben-Shlomo

**Affiliations:** 1 Bristol Medical School: Population Health Sciences, University of Bristol, Bristol, BS8 2PS UK; 2 Southmead Hospital, North Bristol NHS Trust, Bristol, BS10 5NB UK; 3 Erie County Medical Center, Buffalo, NY 14215 USA

**Keywords:** Kidney transplantation, Nephrology, Feasibility, Randomised controlled trial

## Abstract

**Background:**

The UK’s living-donor kidney transplant (LDKT) activity falls behind that of many other countries internationally, with less than 20% of those eligible receiving a LDKT each year. Certain individuals with kidney disease in the UK appear to be particularly disadvantaged in accessing a LDKT; the most socioeconomically deprived people with kidney disease are 60% less likely to receive a LDKT than the least deprived. Improving equity in living-donor kidney transplantation has been highlighted as an international research priority.

**Methods:**

This feasibility trial was designed to determine the feasibility of delivery and acceptability of a multicomponent intervention designed to improve access to living-donor kidney transplantation. The intervention comprises three main components: (i) a meeting between a home educator and the transplant candidate for a dedicated discussion about living-donor kidney transplantation, living kidney donation and potential donors; (ii) a standardized letter from a healthcare professional to a candidate’s potential donors and (iii) a home-based education and family engagement session including two home educators, the transplant candidate and their family. The primary objectives are to establish the feasibility (i) of delivering the developed intervention in existing care pathways and (ii) of undertaking a randomised controlled trial of the intervention. A mixed-methods parallel process evaluation will investigate the acceptability, implementation and mechanisms of impact of the intervention. The trial is based at two UK hospitals: a transplanting hospital and a non-transplanting referral hospital. Individuals are eligible if they are ≥ 18 years old, are active on the kidney transplant waiting list or have been referred for transplant listing and do not have a potential living-donor undergoing surgical assessment. Randomisation will be undertaken with concealed allocation. Participants will be randomly allocated 1:1 to (i) the intervention or (ii) usual care, stratified by site to ensure a balance in terms of local differences. Minimisation will be used to ensure balance in sex, age group and socioeconomic strata, with probability weighting of 0.8 in order to reduce predictability. The primary outcomes are recruitment (% of those eligible and invited who consent to randomisation) and retention (% of participants completing follow-up).

**Discussion:**

Findings will inform the design of a future fully powered, randomised controlled trial to formally evaluate the effectiveness of the intervention at improving equitable access to living-donor kidney transplantation.

**Trial registration:**

ISRCTN Registry ISRCTN10989132 Applied 30/10/20.

**Supplementary Information:**

The online version contains supplementary material available at 10.1186/s40814-023-01241-1.

## Background

A living-donor kidney transplant (LDKT) describes a transplant in which a kidney has been donated from a living person, typically a relative or friend. It is the best treatment in terms of life expectancy for most people with kidney failure [1–7]; in the UK, 90% of adult LDKT recipients are alive 10 years after transplantation, compared to 77% of deceased-donor transplant recipients [7]. Quality of life is better with a transplant compared to dialysis [1–6]. The risks of donating a kidney are very small [8–10]. Mortality in living kidney donation is estimated to be between 0.01 and 0.03% [11–13]. Perioperative complications, such as wound infection and bleeding, occur in about 7.3% of cases [12]. Absolute 15-year incidence of kidney failure for white kidney donors is < 1%, and the quality of life of donors returns to pre-donation levels after donation [5, 14–16]. The cost benefit of kidney transplantation compared to dialysis over a period of 10 years is approximately £20,000 per year for each year that the patient has a functioning transplant [17].

The UK’s LDKT activity falls behind that of many other countries, including the Netherlands and the USA [18]. Only 20% of those listed on the national transplant waiting list receive a LDKT each year [4]. Certain individuals with kidney disease appear to be less likely to receive a LDKT. Socioeconomic deprivation describes the disadvantage of an individual or group relative to others in society, as indicated by people’s education, employment, income and assets [19, 20]. In the UK, socioeconomic deprivation is associated with reduced access to living-donor kidney transplantation [21, 22]; the most socioeconomically deprived people with kidney disease are 60% less likely to receive a LDKT than the least deprived [21].

Improving equity in living-donor kidney transplantation has been highlighted as a UK and international research priority [23, 24]. This feasibility trial follows on from a mixed-methods intervention development study [25]. In this previous work, we developed a multicomponent intervention to address previously identified barriers to living-donor kidney transplantation, focussing on those that appeared to explain the observed socioeconomic inequity in the UK. Four variables have been identified as key mediators of the inequity and thus are targets to the intervention: LDKT transplant knowledge, patient activation, perceived social support and health literacy. Patient activation describes the ‘knowledge, skills and confidence a person has in managing their own health and health care’ [26]. Social support comprises the emotional, physical, practical, informational and relational assistance [27]; perceived social support describes what support an individual perceives is available and may not correlate with true available received social support. Finally, health literacy describes an individual’s ability to obtain, process and understand basic health information needed to make appropriate health decisions [28]. Socioeconomic deprivation is associated with a lack of LDKT knowledge [29, 30], lower levels of ‘patient activation’ [29, 30], perceived low levels of social support [29, 30] and lower health literacy [31]. The developed intervention [25] is described in detail in ‘Methods’ but comprises the following elements:

1. A meeting between a home educator and the transplant candidate — Dedicated discussion about living-donor kidney transplantation and living kidney donation
2. A standardized letter from a healthcare professional to a candidate’s potential donors
3. A home-based education and family engagement session including the two home educators: Home-based education and family engagement

The clinician letter to potential donors is a standard practice in Norway [32] but has never been formally evaluated for effectiveness or for potential harms such as potential donors feeling pressure to donate. The home-based patient and family education intervention has been evaluated in randomised controlled trials (RCTs) in the Netherlands and the USA [33, 34]. In the study from the Netherlands, there were more LDKTs in the experimental group compared with the control group (17/39 vs. 4/41, *p* = 0.003) [33]. In the USA trial, compared to control, more patients in the home-based education group had LDKTs (30.4% vs. 52.4%, *p* = 0.013) [34]. However, the healthcare systems and populations of these countries are not directly comparable to those of the UK. It is not known if intervention delivery is feasible in the NHS, if the intervention is cost-effective in the UK or if it reduces inequity in living-donor kidney transplantation which the larger planned RCT aims to determine. The intervention components have not been evaluated together as a single intervention that targets multiple barriers to living-donor kidney transplantation.

This feasibility trial is designed to gather information on the logistics of delivery, acceptability and risk of harm of the developed intervention and to determine if it would be possible to conduct a full-scale trial. A parallel process evaluation [35] will investigate the acceptability, implementation and mechanisms of impact of the intervention.

### Objectives

The primary objectives are as follows:

- To establish the feasibility of delivering the developed intervention in existing NHS care pathways by diverse practitioners at different hospital sites
- To establish if it is possible to undertake an RCT of the intervention

The secondary objective is to identify ways in which trial processes and intervention delivery could be improved to optimise the future full-scale trial and intervention.

Process evaluation objectives are to assess the following:

- Acceptability of the intervention to transplant candidates, their family and friends and healthcare practitioners
- Acceptability of study procedures to participants and healthcare practitioners

Findings will inform the design of a future fully powered, randomised controlled trial to formally evaluate the effectiveness of the intervention at improving equitable access to living-donor kidney transplantation.

## Methods

### Trial design

**Fig. 1:**
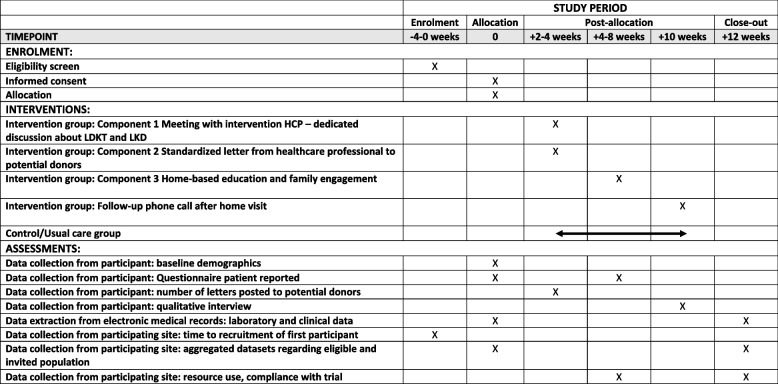
SPIRIT Fig. [36] for schedule of enrolment, interventions and assessments

This trial is a two-arm, parallel group, pragmatic, individually randomised, controlled, feasibility trial of a complex multicomponent intervention, comparing the new intervention with usual care. A cluster RCT was considered, but contamination of controls was not anticipated to be likely, and an individually randomised RCT was more cost-effective. A mixed-methods parallel process evaluation is being undertaken. Figure 1 shows the schedule of enrolment, intervention and data collection assessments as a SPIRIT (Standard Protocol Items: Recommendations for Interventional Trials) 2013 [36] (Fig. 1).

### Study setting and participants

The feasibility trial will run at two UK NHS hospitals: one hospital is a transplanting centre and regional referral centre, and one hospital is a non-transplanting referral centre. Both hospitals serve people with all stages of kidney disease, including those in receipt of kidney replacement therapy and those receiving conservative care. The non-transplanting referral centre does not carry out kidney transplants; patients are referred to two other hospitals for assessment for transplantation and for the transplant operation. The non-transplanting centre carries out initial assessments of potential living kidney donors, but these donors are finally assessed by a transplant centre, which also carries out the donation surgery. Both participating hospitals are urban hospitals but serve both urban and rural areas.

#### Inclusion and exclusion criteria

English-speaking adults (age ≥ 18 years) are eligible if they have the following:

i) Have been referred for/are being assessed for transplant listing AND do not have the following:
  - Active malignancy
  - Signs or symptoms of active cardiac disease (e.g. angina, arrhythmia, New York Heart Association (NYHA) functional class3/4 heart disease, symptomatic valvular heart disease)
  - Chronic intractable systemic infection
  - Active substance addiction/misuse
  - Body mass index (BMI) ≥ 40 kg/m.^2^
  - A transplant MDT prediction that they are unlikely to be suitable following pending investigations (e.g. cardiopulmonary)

Or

ii) Are active on the UK Kidney Transplant only waiting list.

Individuals are assessed for kidney transplantation when they have advanced kidney disease (this includes chronic kidney disease stages 4, 5, 4T and 5T and those in receipt of dialysis therapy). Individuals will be excluded from participation if they:

i) Have a potential living kidney donor undergoing surgical assessment for donation or approved for kidney donation
ii) Lack the mental capacity (as determined by their healthcare team) to consent to participation

At the end of the study, the sites will provide anonymised datasets to the central study team with details of the (i) eligible population at each study site and (ii) the population invited to participate. The sites will share anonymised aggregated datasets only, summarising the number of individuals eligible for participation, the number of individuals invited to participate and, for each dataset, the % female, % White ethnicity, % English Index of Multiple Deprivation (EIMD) decile ≤ 5 and median age. This will enable assessment of those not reached by the study and intervention and allow assessment of the applicability of study findings to the truly eligible population. For nonparticipants, only aggregate data will be shared. Data will be compared against anonymised data on the eligible population at each study site (including socioeconomic status using EIMD [37] deciles), extracted from the UK Renal Registry (UKRR).

### Recruitment

The information technology (IT) lead at the transplanting centre will generate a list of all adults currently active on the UK Kidney Transplant only waiting list, generated from the renal IT system (proton, diproton, vital data). Each study site will be provided with the list of their transplant listed population. This list will be refreshed after 6 months as some initial screen failures may become eligible for participation.

Transplant and living donor coordinators, nephrologists, surgeons and dialysis nurses can approach eligible individuals in person or over the telephone to invite them to participate and will provide them with a patient information sheet. This will explain that the trial will compare (i) extra support with (ii) usual care to see if this alters the chances of getting a kidney transplant. Process evaluation qualitative interviews are planned with patient participants, their family and friends and healthcare professionals (HCPs). Patient participants allocated to the intervention arm will be asked to invite family members and friends to an education and engagement home visit. Family members and friends present at this home visit will be invited to participate in qualitative interviews. HCPs involved in delivering the intervention, contributing to the research study, or those whose practice will be impacted by the intervention will be invited to participate in qualitative interviews. HCPs will be identified by their existing participation in the research study and clinical positions within participating study site.

### Consent

Written consent to trial participation will be requested from eligible individuals by research nurses at participating sites. Only patients with the mental capacity to make this decision will be able to enter the trial. If a patient who has provided consent to participation loses capacity to provide ongoing consent to participation, they will receive no further trial intervention, assessments or contacts from the research team. Data on outcomes for these individuals will be extracted from the national databases and registries (UK Renal Registry, NHS Blood and Transplant, NHS Digital). Consent for process evaluation qualitative interviews will be requested at the time of interview. If the interview is undertaken face to face, written consent will be requested. If the interview is undertaken over the telephone, consent will be audio-recorded, and the researcher who completes the consent form will sign to confirm the consent has been recorded. The recording of the consent will be transcribed along with the interview.

### Allocation

Participants will be randomised to either (i) the intervention arm or (ii) usual care. Research nurses will undertake randomisation of eligible individuals with concealed allocation using Internet-based Sealed Envelope™ software using minimisation. Participants will be allocated 1:1 to the intervention arm or to the usual care arm, stratified by site to ensure a balance in terms of local differences. Minimisation will be used to ensure balance in sex, age group (≤ 55 years vs > 5 5 years) and socioeconomic strata (EIMD [37] 2019 deciles 1–5 vs deciles 6–10). We will use minimisation with probability weighting of 0.8 in order to reduce predictability. Due to the nature of the intervention, participants and those administering the intervention cannot be blinded to allocation.

### Intervention

Two HCPs will deliver the intervention at participating sites. The participant should receive their first contact from an intervention HCP within 1 week of consent and referral. This will be over the telephone. This first contact will cover initial introductions and planning a date for a first ‘face-to-face’ meeting. Figure 1 shows the intervention components and timings. Intervention components 1 and 2 happen at the same meeting.

#### Intervention component 1: meeting between intervention HCP and transplant candidate — dedicated discussion about living-donor kidney transplantation and living kidney donation

The participant will meet with an intervention HCP within 2–4 weeks of recruitment. The first meeting should be undertaken in person, but if the participant requests it, the first meeting can be undertaken over the telephone or a virtual platform. The HCP will discuss living-donor kidney transplantation and living kidney donation with the participant. Participants will be informed about an educational animation video presenting an overview of living-donor kidney transplantation (adapted for UK use from animations developed by Dr. Liise Kayler Health Resources and Services Administration (HRSA) funded work in the USA [38]). The animation will be shown and links shared if the participant wishes. The HCP will ascertain the participant’s close relationships with relatives and friends and the estimated age, sex and health of these individuals. This information will be recorded and illustrated in a sociogram. No personal identifiers will be recorded in the sociogram. They will discuss the initial suitability of relatives and friends as potential living donors.

#### Intervention component 2: standardised letter from healthcare professional to candidate’s potential donors

The intervention HCP will provide the participant with a copy of two intervention resources: a ‘Clinical information letter’ and information leaflet ‘Donating one of your kidneys’. The intervention HCP will agree with the participant to which family members and close friends letter and information on living kidney donation will be posted. The sharing of the letter and information with family and friends will be facilitated by the participant. The participant will address envelopes for posting to selected family members and close friends, but postage will be paid by the study. The intervention HCP will not request access to or record any contact details of the family and friends of the participant. Alternatively, the participant can take home the envelopes and address these at home, or they can distribute to family and friends. The intervention HCPs will also take the ‘Clinical information letter’ and ‘Donating one of your kidneys’ leaflet to the home visit (Intervention component 3: home-based education and family engagement) for sharing with family members and friends present.

The intervention HCP will inform the participant about the third component of the intervention: the home education and engagement visit. The intervention HCP will explain the proposed content of the home visit and will agree with the participant (i) which friends and family members will be invited to the home visit, (ii) where the visit will take place (home preferred location but participants may ask for an alternative location such as a community space, a hospital meeting room), and (iii) a number of possible dates for the home visit in the next month. If necessary, the home visit can be conducted over a virtual platform and/or family and friends could join the meeting virtually. At the end of the first session, the intervention HCP will record the number of individuals that the participant wishes to invite for the second session.

#### Intervention component 3: home-based education and family engagement

The home visit will be undertaken by two intervention HCPs who will deliver the education and engagement package approximately 2–4 weeks after the first meeting. The home visit can take place at any day and time that suits the participant and their guests; this includes evenings and weekends. In exceptional cases, a second home visit may be required.

The home education and engagement session will cover the following: kidney disease (specific to the participant’s disease); dialysis; transplantation — deceased-donor and living-donor transplantation; living kidney donation; and open discussion.

The HCPs will share their work telephone number and work email addresses with participants so that they can be contacted for further advice and support if required at any time during the trial. If requested, a second home visit covering all or part of the content of the first visit can be delivered.

#### Intervention modifications

This study is a feasibility trial to finalise the intervention before undertaking a full-scale RCT. The parallel process evaluation will mean data is collected on the acceptability of the intervention components alongside delivery. This will be used to make minor changes to the intervention components as part of an iterative development of the resources to optimise acceptability and reach of the intervention prior to evaluation in the full-scale RCT. Suggested changes to intervention resources will be considered using the person-based approach [39] as in intervention development [25].

### Usual care

In this pragmatic trial, all treatment delivered as part of usual care will continue for both trial arms. Usual care typically comprises general renal replacement therapy education (providing education on dialysis modalities, transplantation and conservative care). This education may be provided in a ‘low clearance/options for kidney care clinic’ or provided in the home environment. Usual care does not include specific education for patients or their families on living-donor kidney transplantation or living kidney donation. Usual care does not involve contacting family and friends with information about kidney donation. No restrictions on concomitant treatments are specified.

### Outcomes

The primary and secondary outcomes and outcome measures are detailed in Table 1.

**Table 1:** Primary and secondary outcomes and measures

	**Outcomes**	**Timepoint(s) of evaluation of outcome measures**
Primary	•Recruitment •Retention	•Recruitment at 0 week: % of those eligible and approached who consent to randomisation at invitation •Retention at 4–8 weeks: % completing follow-up questionnaire 4–8 weeks after baseline questionnaire
Secondary	•Size of the eligible participant population •Participant and healthcare professional adherence to the intervention/trial •Fidelity of delivery of the intervention •Acceptability of the intervention and trial methods •Barriers and facilitators to intervention implementation in different settings •Estimates of the effect of the intervention on possible mediators of the intervention •Assessing linkage to UKRR data regarding planned outcome for later main trial (receipt of a LDKT) •Impact on existing healthcare delivery and potential for the intervention to become a normalised part of everyday care •Cost drivers	•Size of the eligible population screening at − 4 to 0 weeks •Adherence to intervention/trial and fidelity of delivery of the intervention assessed at 4–8 weeks — % of participants in intervention arm who receive the various intervention components; number of letters sent to potential donors per participant in intervention arm; % of participants who complete questionnaires at baseline and 4–8 weeks after baseline; number of protocol deviations reported; observed fidelity of intervention delivery •Acceptability of intervention and trial methods at 10 weeks — assessed through qualitative interviews •Barriers and facilitators to recruitment — measured by time to green light at each site and time to recruitment of first participant, assessment of screening log and qualitative interviews with participants, nonparticipants and healthcare professionals •Estimates of the effect of the intervention on possible mediators of the intervention at 4–8 weeks — measured through questionnaires measuring patient activation, social support, LDKT knowledge and health literacy at baseline and 4—8 weeks later • Assessing linkage to UKRR data regarding planned outcome for later main trial (receipt of a LDKT), assessed after closure of trial recruitment •Impact on existing healthcare delivery measured through qualitative interviews with HCPs after 3 months of study being active at study site • Cost drivers measured at feasibility trial completion

The primary outcome for the later full-scale RCT will be receipt of a LDKT. Secondary outcomes will include the number of potential donors undergoing assessment, the proportion of participants receiving a LDKT, participant patient activation, participant-perceived social support, participant transplant/donation knowledge and cost-effectiveness data.

### Sample size

On average each renal unit in the UK has approximately 75 patients (range 20–250) active on the national kidney transplant waiting list [40]. The proportion of those listed who do not have a potential living-donor under assessment will be determined in this work. If 80% of those listed do not have a living donor under review, the eligible population at an ‘average’ site will be approximately 60 individuals. We will recruit from 2 centres, aiming for a target sample size of 60 patients (30 in each arm). This will allow us to estimate a recruitment rate of 50% with a 95% confidence interval of 41 to 59%. In the 60 randomised patients, we will be able to estimate a true rate of follow-up at 1 year of 70% with a 95% confidence interval of 58–82%.

### Data collection

Trial participants will have two standard study contacts and one optional qualitative contact:

1. Baseline
2. Follow-up: Questionnaire completion 1–3 days after the home visit or for those not receiving the intervention in the control arm, 4–8 weeks after the baseline assessment.
3. Qualitative: After the home visit, some purposively sampled patient participants will be invited to consider participating in qualitative interviews.

Clinical and laboratory data will be extracted from hospital medical records by the research nurses. The data collected are reported in hospital medical records and do not require interpretation. Patient-reported outcome data will be collected through questionnaires self-completed by participants. Participant experience of the trial and intervention will be collected through qualitative interviews. Compliance with the intervention will be recorded by the intervention HCPs against a checklist.

#### Baseline data

Demographic, social, clinical and patient reported data will be collected by research nurses at the baseline visit (following consent and prior to randomisation) (Table 2). No blood or urine tests are required other than those that are being performed as part of routine care.

**Table 2:** Summary of baseline data collection for the feasibility randomised controlled trial

Demographics	Age, sex, ethnicity, religion, marital status + / − children, employment status, education level, housing tenure, EIMD 2019 score, distances lived from kidney hospital and transplanting hospital, car ownership, alcohol consumption, smoking history
Clinical	Primary renal disease, date of current transplant listing, comorbidities, current stage of kidney disease (pre-emptive, haemodialysis, peritoneal dialysis), body mass index (BMI)
Patient reported	Renal and transplant knowledge (R3K-T [41]), social support (ISEL-12 [42, 43]), patient activation (PAM13 [26, 44]), health literacy (SILS [45, 46]), transplant preference, transplant beliefs [47], experience talking to family and friends about kidney disease and living donation (binary response questions), quality of life (EQ-5D-5L [48])
Laboratory	Blood group, tissue type at HLA-A, B and DR, matchability (levels 1–10), % calculated reaction frequency
Family and social network	Draw sociogram/social network diagram — containing information on all close relationships with relatives and friends and the estimated age, sex and health of these social contacts

#### Follow-up data

Figure 1 outlines the planned data collection timepoints. Data will be entered directly onto online electronic case report forms (eCRF) and patient participant questionnaires (Table 3).

**Table 3:** Summary of follow-up data collection for the feasibility randomised controlled trial

		Time after baseline
Clinical	Current transplant listing status (active, suspended, removed, transplanted. If transplanted, the date and type of transplant will be recorded), comorbidities, current stage of kidney disease (pre-emptive, haemodialysis, peritoneal dialysis), date of death (if this has occurred), cause of death (if this has occurred), BMI	12 weeks
Laboratory	Matchability, % calculated reaction frequency	12 weeks
Resource use	Number of donors undergoing assessment	12 weeks
Compliance with and acceptability of trial	Number of living donation letters posted to potential donors; number of home visits by intervention healthcare professional (HCP); content compliance (quantitative checklist for home visit content)	4–8 weeks
Patient reported	Renal and transplant knowledge (R3K-T [41]); social support (ISEL-12 [42, 43]); Patient Activation (PAM13 [26, 44]); health literacy (SILS [45, 46]); transplant preference; transplant beliefs [47]; experience talking to family and friends about kidney disease and living donation (binary response questions); quality of life (EQ-5D-5L [48])	4–8 weeks

Standardised tools are being used as follows:

- Demographics: EIMD 2019 score [37].
- Patient-reported mediator and outcome measures: Renal and transplant knowledge (R3K-T) [41], social support (ISEL-12) [42, 43], patient activation (PAM13) [26, 44], health literacy (SILS) [45, 46], transplant preference, transplant beliefs [47], experience talking to family and friends about kidney disease and living donation (binary response questions) and quality of life (EQ-5D-5L [48]).

Participants who have been recruited to the trial can choose to withdraw from their allocated treatment. If they withdraw from treatment, they can continue to consent to follow-up assessments or withdraw from both treatment and follow-up assessments. Data already collected will be kept, and clinical outcomes from hospital records and UKRR and NHS blood and transplant registries will be extracted if the participant has consented to this. Participants who withdraw from follow-up assessments will have no further contact from the research team regarding the trial.

#### Outcome data

At the point of consenting to take part in the RCT, all participants will be asked to consent to linkage to existing healthcare databases held by the UKRR and NHS Blood and Transplant. These databases can provide data on commencement of kidney replacement therapy, date and type of kidney transplant, date and cause of death, if this information is missing from the medical records. This will enable follow-up of clinical outcomes for participants who lose capacity to continue providing consent or are lost to follow-up, including those who move to a renal unit not participating in the trial.

### Mixed-methods process evaluation

A process evaluation will be used to study how the intervention is implemented and may provide information on contextual factors that affect the intervention. It will also provide information about the uniformity of delivery of the intervention to different participants in different locations, where the ‘same’ intervention may be delivered and received in different ways. The integrated qualitative research will provide a more in-depth understanding of how the trial treatments and procedures are being delivered and received in practice.

Process evaluation will be undertaken by the chief investigator (CI) and research nurses using the following mixed methods:

- Semi-structured qualitative interviews with participant and non-participant transplant candidates and family members to understand experience and acceptability of intervention and with practitioners on experiences of delivery
- Mixed-methods observations of at least two home visits at each study site, assessing fidelity of delivery against a quantitative checklist and making qualitative observation field notes
- Quantitative data collection regarding patient participation aggregate data from participating sites on eligible population and invited population, including % of those eligible who were invited to participate and % of those invited who agreed to participation, were recruited

Fidelity of delivery of the first meeting part of the intervention will be assessed using implementer self-report against a structured checklist and qualitative interviews with participants and implementers. Fidelity of the home visits will be assessed through structured observations with a quantitative checklist, qualitative observation notes and participant and implementer qualitative interviews.

Process information will be documented in the compliance log. For participants in the intervention arm, the research nurse, an intervention HCP or CI will use the quantitative intervention delivery checklist, recording the delivery of components of the intervention. For participants in the control, arm compliance will be recorded at the end of the study by the research nurse recording whether the participant received any elements of the intervention from another source. We will explore the reason for not complying with treatment as recorded in the compliance log and investigated through qualitative interviews. If participants who are noncompliant with their allocated treatment arm do not respond to attempts to collect patient-reported outcome data, outcome data that can be extracted from medical records and national registries will be used.

Qualitative interviews will be used to investigate the following:

i) **Reasons for nonparticipation:** Individuals who decline to participate in the trial will be asked if they will be willing to explain why they do not wish to participate, over a telephone interview.
ii) **Experience of the intervention and trial:** Following delivery of the intervention (clinical invitation letters to family and friends and the home education and engagement session), patient participants, family and friends present at the home visit and practitioners will be invited to an in-depth qualitative interview which can be undertaken in person or over the telephone. These interviews will investigate the acceptability of both the intervention and trial methods and will be used to investigate ways of tailoring and optimising the intervention resources for future trials. Participants will be purposively sampled aiming for diversity in age, sex, ethnicity, socioeconomic status and primary renal disease. Participation in the qualitative interviews is optional.

Example interview topic guides are provided as supplemental material.

### Analytical methods

Analysis and reporting will be in line with the CONSORT (Consolidated Standards of Reporting Trials) extension for randomised pilot and feasibility trials [49]. Patient-reported outcome scores from questionnaire data will be calculated based on the developers’ scoring manuals, and missing and erroneous items will be handled according to these instructions. Continuous measures will be presented as means and standard deviations or medians and ranges depending on their distribution. Categorical data will be presented as frequencies and proportions, with 95% confidence intervals.

The study will assess the following primary outcomes:

- Recruitment: % of those eligible and approached who consent to randomisation at invitation.
- Retention: % completing follow-up questionnaire approximately 4–8 weeks after baseline questionnaire.

We will describe the primary outcome variables across socioeconomic position quantiles.

Descriptive statistics will be used to report the secondary outcomes of interest.

Qualitative interviews will be analysed using inductive thematic analysis, as described by Braun and Clarke[50]. Transcripts will be read twice to gain familiarisation and sections of text coded by assigning descriptive labels. Codes will be grouped based on shared properties and themes identified. All transcripts will be independently coded by two researchers, the CI and another qualitative researcher. Codes will be discussed to maximise rigour and reliability, to identify areas of discrepancy and to refine the coding system. NVivo software will be used to aid analysis. Data collection and analysis will be conducted concurrently, employing an iterative approach. The sample size for the qualitative phase will be determined by reaching theoretical theme saturation [51].

### Data management

Clinical data will be stored using REDCap [52]. REDCap is a secure, web-based electronic data capture system designed for the collection of research data. Although the system has been developed (and it is supported) by Vanderbilt University, the University of Bristol (UoB) has set up its own infrastructure to host the REDCap application so that all elements reside within UoB. REDCap is used solely for anonymised clinical data linked by a participant ID. Data are stored in a secured UoB server subject to standard UoB security procedures. The full database is backed up daily. Additionally, changes are logged every 5 min. A disaster/recovery plan is in place as part of the service-level agreement (SLA) we have with IT services. Data entry can be performed by accessing the REDCap application directly or via surveys. In order to access the application directly, users will be added to the system following request to the CI. It is the CI’s responsibility to add the user to a specific project and role.

Qualitative data will be collected as encrypted digital audio files and then transcribed into word files through secure University of Bristol-approved transcription services. Anonymised transcripts will be uploaded to NVivo software for analysis, and audio files will be destroyed. No one other than the CI and the transcription service will be allowed access to the qualitative audio files due to the risk of participant identification.

### Decision whether to progress to full RCT

A quantitative dashboard with green/amber/red thresholds is proposed to guide decision-making following this study on progression to a full-scale RCT (Table 4). Achieving all green targets would almost certainly mean proceeding to the full trial, whereas achieving predominantly red targets would almost certainly indicate that a full-scale RCT is not feasible.

**Table 4:**
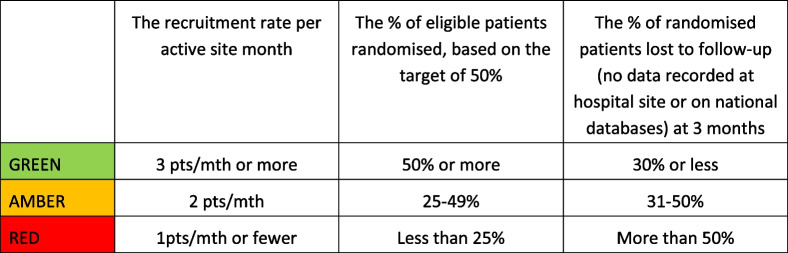
Green/Amber/Red progression criteria

Analysis of questionnaire responses and thematic analysis of the qualitative interview data regarding acceptability of the intervention and trial processes will further assist with decision-making on progression. Progression will only occur if the majority of participating renal patients and their families report that the intervention and trial processes are acceptable.

### Monitoring

The Trial Management Group (TMG) will comprise of the CI (PKB), coinvestigators (YBS, FC, SM), the intervention HCPs and patient representatives. The TMG will meet approximately once each month in the first 6 months, then 6 monthly to review progress, with potential for additional ad hoc meetings, as required/indicated. These meetings will be chaired by the CI. Participants will have the opportunity to join the meetings in person or virtually.

The Trial Steering Committee will be chaired by an independent member and comprise a minimum of four additional independent members (covering expertise in transplantation and clinical trials and including people who have received a kidney transplant or donated a kidney). The CI (PKB) will also be a member of the TSC. Observers may also attend, as may other members of the TMG or members of other professional bodies, at the invitation of the chair. The TSC will meet for the first time by month 6 of the trial and then at least every 6 months until the trial closes. For this feasibility trial, the TSC will also have responsibility for data monitoring. For discussions regarding data monitoring, the CI and other members of the TMG will be asked to leave the room so that the data monitoring is only undertaken by the independent members of the TSC, without the influence of those involved in the delivery of the trial.

### Harms

Due to the nature of advanced kidney disease and its treatments, serious adverse events (SAEs) would be expected to occur throughout the course of the disease and therefore this study. These expected SAEs include the following:

- Abnormal electrolyte and haematological laboratory results that can be explained directly or indirectly by their advanced kidney disease
- Hospital admissions — elective and emergency — that can be explained directly or indirectly by their advanced kidney disease
- Hospital admissions for elective procedures with the exception of a kidney transplant
- Infections and cardiovascular events that can be explained directly or indirectly by their advanced kidney disease
- Commencement of dialysis
- Death that can be explained directly or indirectly by their advanced kidney disease

These expected SAEs do not require reporting to the sponsor but will be recorded in the participant’s medical records. However, anything not in the above list, or anything the principal investigator or CI deems unexpected, must be reported to the sponsor.

All reportable SAEs occurring from the time of consent until 30 days after the end of the trial must be documented and emailed securely to the sponsor within 24 h of the research staff becoming aware. The CI will report any SAE that is related to the research procedures and unexpected to the research ethics committee and the sponsor within 24 h of becoming aware of the event.

For each SAE, the following information will be collected as follows:

- Full details in medical terms and case description
- Event duration (start and end dates, if applicable)
- Action taken
- Outcome
- Seriousness criteria
- Causality (i.e. relatedness to trial/intervention), in the opinion of the investigator
- Whether the event would be considered expected or unexpected

Any change of condition or other follow-up information should be securely emailed to the sponsor as soon as it is available or at least within 24 h of the information becoming available. Events will be followed up until the event has resolved or a final outcome has been reached.

Each SAE must be reported separately. Any change of condition or other follow-up information relating to a previously reported SAE should be documented and emailed securely to the sponsor as soon as it is available or within at least 15 days of the information becoming available to the research team. Events will be followed up until the event has resolved or a final outcome has been reached.

All other adverse events will be reported to the CI by sites via an online AE form, and a summary report will be submitted to the TSC on a regular basis.

### Auditing

All trial-related documents will be made available on request for monitoring and audit by the University of Bristol, the Research Ethics Committee and available for inspection by other licensed bodies. Monitoring and audits undertaken by the University of Bristol, under their remit as sponsor, or individuals appointed responsibility for monitoring on their behalf, will ensure adherence to good clinical practice and the UK policy framework for health and social care research. Remote monitoring will be conducted based on information submitted by sites and analysis of the trial database. Site visits will then be initiated using a risk-based approach.

The University of Bristol Research Governance Department also regularly reviews its research portfolio. The University of Bristol has a service-level agreement with University Hospitals Bristol NHS Foundation Trust, whereby the trusts monitor 10% of the university’s sponsored studies. University Hospitals Bristol NHS Foundation Trust has a standard operating procedure (SOP 014 Monitoring and Oversight of Research) available at http://www.uhbristol.nhs.uk/research-innovation/for-researchers/run-a-study-and-closedown/monitoring/.

### Confidentiality

The University of Bristol will be the data custodian. All data held in Bristol will conform to the University of Bristol Data Security Policy and in compliance with the Data Protection Act 1998. Data will be managed according to the University of Bristol’s Research Data Management policy: http://www.bristol.ac.uk/research/environment/governance/research-data-policy/.

### Access to data

Anonymous research data will be stored securely and kept for future analysis. Data will be uploaded to the University of Bristol’s Research Data Repository: https://data.bris.ac.uk/data/.

The final qualitative and quantitative dataset will be accessible by the CI, the coinvestigators, the trial statistician and the TSC. Local PIs will not have access to final dataset unless formally requested and approved by the TSC. The anonymised qualitative data transcripts will be suitable for sharing with other researchers who may wish to undertake a thematic synthesis or analyse the interviews using a different methodology to the one used in this study. Consent for the sharing of the interview transcripts to other researchers will be explicitly sought from interviewees prior to the interview, and this confirmed with the interviewee following the interview. Transcripts will be anonymised with all personal identifiers and possible identifiers redacted. This includes details that may identify other people mentioned in the interview, e.g. clinicians and family members. Participants will be informed that transcripts will be stored in accordance with the Data Protection Act [53], and they will be asked to confirm in writing that they have understood this. If consent for sharing is not provided, the data will be used by the primary research team only. Due to personal issues being discussed, we cannot rule out the risk of reidentification therefore as a double safeguard access to these transcripts will be controlled. Requests for controlled data through the University of Bristol are referred to an appropriate Data Access Committee (DAC) for approval, before data can be shared with bona fide researchers, after their host institution has signed a Data Access Agreement. The university’s DAC comprises the following: assistant director of research services (library), information rights officer (FOI, data protection), head of research governance (ethics), assistant director IT services (data security), research contracts (if commercially sensitive), academics, e.g. the PI. The procedure for accessing data can be found here: https://www.bristol.ac.uk/staff/researchers/data/accessing-research-data/.

If the DAC grants access to the data, a University of Bristol Data Access Agreement is drawn up and signed by the applicant, their host institution and the University of Bristol. The University of Bristol’s Research Data Service will oversee this.

The feasibility study has not been designed to allow meaningful analysis of the quantitative outcome data at this stage but rather to inform the design of a later randomised controlled trial. However, there have been two similar trials in other countries [33, 34], and data might be requested for meta-analysis. Data will be made available to other researchers on request after a period of exclusive use by the researchers of 5 years.

### Dissemination policy

The results of the study will be published in academic journals, and all participants will be offered a plain English summary of the main findings of the study. The main trial report will be prepared and published with reference to the CONSORT 2016 guideline extension to randomised pilot and feasibility trials [49]. Qualitative research reports will be prepared and published with reference to the COREQ guidelines [54]. Work will be submitted for presentation at kidney and transplant national and international conferences.

A final report will be prepared for the funders (The Wellcome Trust and Kidney Care UK) which includes the UK renal patient charity Kidney Care UK. The charity and representatives are active on social media and have established channels for communicating the progress and findings of the study to patients such as through regular newsletters, a network of kidney patient associations and annual meetings. Research findings will be publicised in collaboration with the University of Bristol’s Communications and Media team.

Authorship on manuscripts arising from the trial will be on an individual authorship basis (rather than group authorship basis) with inclusion based on the recommendations of the International Committee of Medical Journal Editors. This protocol was written with reference to the SPIRIT 2013 [36] and CONSORT 2016 guideline extension to randomised pilot and feasibility trials [49].

## Supplementary Information

**Additional file 1.** Topic guides for interviews with: i) renal and transplant healthcare professionals, ii) family and friends who attended home visits, iii) non-participants, and iv) patient participants.

## Data Availability

Not applicable — protocol only.
