# Supplementary material for: Investigating strategies to improve AccesS to Kidney transplantation (the ASK trial): a protocol for a feasibility randomised controlled trial with parallel process evaluation
Source: Pilot Feasibility Stud. 2023 Jan 20;9:13. doi: 10.1186/s40814-023-01241-1 (PMC9854094; doi:10.1186/s40814-023-01241-1)
Supplement: Supplementary file 1 — Additional file 1. Topic guides for interviews with: i) renal and transplant healthcare professionals, ii) family and friends who attended home visits, iii) non-participants, and iv) patient participants. [file 40814_2023_1241_MOESM1_ESM.zip › The ASK trial - health worker qualitative topic guide v1.0R2.docx]

**Topic guide for interviews with renal and transplant healthcare professionals**

| **Topic** | **Questions** |
| --- | --- |
| Views towards trial | What did you think about the trial? Why?  Did your thoughts change as the trial progressed?  Did you feel you had enough information about the study before it started? What did you want to know that you weren’t told? |
| Involvement in trial recruitment | Were you involved in identifying people eligible for the trial? Did you decide any eligible patients were not suitable for recruitment? How did you make these decisions?  Was there anyone you thought might benefit from the trial who wasn’t eligible? |
|  | Were you involved in recruiting patients to the trial? How did you describe the trial to participants? What did you say the point of the study was? What reactions did you get from patients? Did patients who agreed to take part explain why they had made this decision? Did any patients who declined to participate tell you why they had made this decision? |
|  | Were you asked by any patients you saw in your routine clinical practice about the trial? |
| Involvement in direct letter and information to family and friends | Were you involved in discussing with participants their family trees? How did you find this? What issues were raised during these discussions? Did you feel able to answer any questions at this stage? |
|  | How did intervention arm participants respond to the plan to contact their family and friends directly with information about donation? Did any participants ask for a different or tailored approach? If so, how? |
| Involvement in home visits | Were you involved in delivering or observing any home visits? How did you find these? What was difficult/challenging? What, if anything, would you change about the content of the home visits? Were there any issues that were raised that you couldn’t address (either related to kidney transplantation, donation or any other issue)?  If you were involved in delivering or observing more than one home visit, did you observe any differences in the content of the different home visits? Investigate differences if reported. |
|  | What reactions did you observe to the home visit? Did you receive any informal feedback on the home visits from participants (patients/family/friends)? |
|  | Who do you think should deliver the home visits? |
|  | Overall what did you think of the home visit approach? |
| Controls requesting intervention | Did any people in the control group ask you for more information about the intervention? |
| Non-participants | Did any patients outside of the trial asked you about the trial and the extra-support being offered? How did you respond? |
| NHS support | Do you think that your NHS trust and colleagues would be supportive of the interventions delivered in this trial? |
