# Supplementary material for: Investigating strategies to improve AccesS to Kidney transplantation (the ASK trial): a protocol for a feasibility randomised controlled trial with parallel process evaluation
Source: Pilot Feasibility Stud. 2023 Jan 20;9:13. doi: 10.1186/s40814-023-01241-1 (PMC9854094; doi:10.1186/s40814-023-01241-1)
Supplement: Supplementary file 1 — Additional file 1. Topic guides for interviews with: i) renal and transplant healthcare professionals, ii) family and friends who attended home visits, iii) non-participants, and iv) patient participants. [file 40814_2023_1241_MOESM1_ESM.zip › The ASK trial - home visit qualitative topic guide v1.0R2.docx]

**Topic guide for interviews with family and friends who attended home visit**

| **Topic** | **Questions** |
| --- | --- |
| Communication on LDKT before home visit | Before the home visit, what did you know about your family member’s/friend’s kidney disease? Did you know they could have a kidney transplant? Did you know anything about living kidney donation? Discuss sources of information. |
|  | Before the home visit had you ever had any conversations with your family member/friend about kidney donation? If yes, how did you find these? Were they difficult? Probe as to any discomfort, difficulty, anything that made/would make such conversations easier?  Discuss who initiated these conversations, what was discussed, and outcome. |
| Experience of letter | Were you sent/given a letter from the hospital about kidney donation? If yes, how did you feel about receiving this? Discuss any positive/negative responses. Did you receive this through the post or were you given it in person? Did you discuss or share the letter with anyone else? Did you discuss it with your relative/friend with kidney disease? |
|  | What did you think about the information leaflet that was sent to you? Interviewer and interviewee to each have a copy of information sheet to discuss. Discuss any suggested changes.  Did you look for any further information e.g. follow links to websites? |
| Experience of home visit | How did you hear about the home visit? How were you invited to attend? How did you feel about this? What did you think would happen at the home visit? How do you think it could be better described to family and friends? Did you discuss the invitation with any other family or friends? How did you decide whether to attend the home visit or not? |
|  | How did you find the home visit? Discuss content and communication tools used (patient stories, animations, leaflets). How did the home visit make you feel? Did you learn anything from the home visit? What would you change? |
| Experiences since home visit | What happened after the home visit? Immediately and over the following weeks.  How did you feel about what had been discussed? Discuss personal reaction and reactions from other family and friends.  Discuss subsequent conversations: did you discuss the meeting with anyone who was at the meeting/anyone who wasn’t at the meeting?  Did you do anything different as a result of the meeting? Probe if talked to family member/friend about donation. Living kidney donation is discussed at the home visit.  After the home visit did you think about kidney donation? |
| Experience of research | How did you feel about experiencing these things (letter, information sheet, home visit) which were delivered as part of a research study? |
| Other comments | Is there anything else you’d like to share about your experience? |
