# Supplementary material for: Investigating strategies to improve AccesS to Kidney transplantation (the ASK trial): a protocol for a feasibility randomised controlled trial with parallel process evaluation
Source: Pilot Feasibility Stud. 2023 Jan 20;9:13. doi: 10.1186/s40814-023-01241-1 (PMC9854094; doi:10.1186/s40814-023-01241-1)
Supplement: Supplementary file 1 — Additional file 1. Topic guides for interviews with: i) renal and transplant healthcare professionals, ii) family and friends who attended home visits, iii) non-participants, and iv) patient participants. [file 40814_2023_1241_MOESM1_ESM.zip › The ASK trial - non-participant topic guide v1.0R2.docx]

**Topic guide for interviews with non-participants**

| **Topic** | **Questions** |
| --- | --- |
| Introduction and background | Please can you tell me a bit about how you found out you had kidney problems. Discuss what treatment options were discussed, how and by whom. |
|  | Before you were asked to take part in this trial did you know you could have a living-donor kidney transplant? Had you talked to your family or close friends about the possibility of having a kidney transplant? Was/Is this difficult? If yes, what makes it difficult? Did anything make it easier? |
| Initial approach and recruitment | How did you feel when you were first asked to take part in the trial? |
|  | What did you think of the information sheet given at the time? Interviewer and interviewee to each have a copy of information sheet to discuss. Did you feel you had enough information to decide whether to take part? What information would you have liked to have had that you didn’t have? Did you have any questions that weren’t answered? |
|  | What influenced your decision to not take part? Did you discuss taking part with anyone else? Probe reasons for non-participation. |
|  | Is there anything that could have changed your decision to take part in the research study? |
| Participation in research | Have you been asked to take part in a research study before? Probe if participated or not. |
|  | Would you be interested in taking part in future research studies? What sort of studies would you be interested in being approached for? |
| Behaviour change | Has the information provided in the information sheet led to you having any conversations with family and friends about kidney donation? |
|  | Has the information provided in the information sheet led to you having any conversations with your doctor about kidney transplantation? |
|  | Has anyone offered to donate a kidney to you since you started taking part in this trial? How did you respond to this offer? Have you asked anyone to consider donating a kidney to you? |
| Other comments | Is there anything else you’d like to share about your experience? |
