# Supplementary material for: Investigating strategies to improve AccesS to Kidney transplantation (the ASK trial): a protocol for a feasibility randomised controlled trial with parallel process evaluation
Source: Pilot Feasibility Stud. 2023 Jan 20;9:13. doi: 10.1186/s40814-023-01241-1 (PMC9854094; doi:10.1186/s40814-023-01241-1)
Supplement: Supplementary file 1 — Additional file 1. Topic guides for interviews with: i) renal and transplant healthcare professionals, ii) family and friends who attended home visits, iii) non-participants, and iv) patient participants. [file 40814_2023_1241_MOESM1_ESM.zip › The ASK trial - patient qualitative topic guide v2.0 cleanR2.docx]

**Topic guide for interviews with patient participants**

| **Topic** | **Questions** |
| --- | --- |
| Introduction and background | Please can you tell me a bit about how you found out you had kidney problems. Discuss what treatment options were discussed, how and by whom. |
|  | Before you were asked to take part in this trial did you know you could have a living-donor kidney transplant? Had you talked to your family or close friends about the possibility of having a kidney transplant? Was/Is this difficult? If yes, what makes it difficult? Did anything make it easier? |
| Initial approach and recruitment | How did you feel when you were first asked to take part in the trial? Have you been asked to take part in a research study before? Discuss outcomes if asked. |
|  | What did you think of the information sheet given at the time? Interviewer and interviewee to each have a copy of information sheet to discuss. Did you feel you had enough information to decide whether to take part? What information would you have liked to have had that you didn’t have? Did you have any questions that weren’t answered? |
|  | What influenced your decision to take part? Did you discuss taking part with anyone else? |
| Randomisation – feeling on allocation | How did you feel about the computer system deciding if you got the extra support or not? |
|  | How did you feel when you found out you had been allocated to receive extra support/to receive usual care and no extra support? |
| Experience of control arm – experience and effect of participation | How did you feel about being asked to fill out the questionnaires? Were they difficult to fill out? |
|  | Did you talk to any family or friends about taking part in the research trial? What did you tell them? Did taking part in the trial help you to have any conversations with family and friends about kidney donation? Did you do anything to try to get the extra support being offered to other people in the trial from your own doctors? e.g. request info be sent to family members, request home visit. |
| Experience of intervention arm – experience and effect of participation | How did you find discussing your family tree/genogram with the nurse specialist? Did you find anything helpful about this? Did discussing this result in you speaking to any family/friends about donation? |
|  | How did you feel about letters being sent to family members? Were these posted or did you give them in person? What reactions did you get from family and friends? Discuss any positive/negative responses. What did you think about the information sent to your family and friends? Interviewer and interviewee to each have a copy of information sheet to discuss. |
|  | How did you feel about inviting people to your home for the home visit? How did you do this? Did you ask or did someone else ask for you? What were the reactions of family and friends to this? |
|  | How did you find the home visit? Discuss content and communication tools used (patient stories, animations, leaflets). What would you change about the home visit? |
|  | What happened after the home visit? How did you feel? Discuss personal reaction and reactions from family and friends. Discuss subsequent conversations: did you discuss the meeting with anyone who was at the meeting/anyone who wasn’t at the meeting? |
| Experiences since intervention | Has anyone offered to donate a kidney to you since you started taking part in this trial? How did you respond to this offer? Have you asked anyone to consider donating a kidney to you? |
| Other comments | Is there anything else you’d like to share about your experience? |
